# Supplementary figures and images for: Hypoglycemic Effects and Mechanisms of Buckwheat–Oat–Pea Composite Flour in Diabetic Rats
Source: Foods. 2022 Dec 6;11(23):3938. doi: 10.3390/foods11233938 (PMC9739861; doi:10.3390/foods11233938)

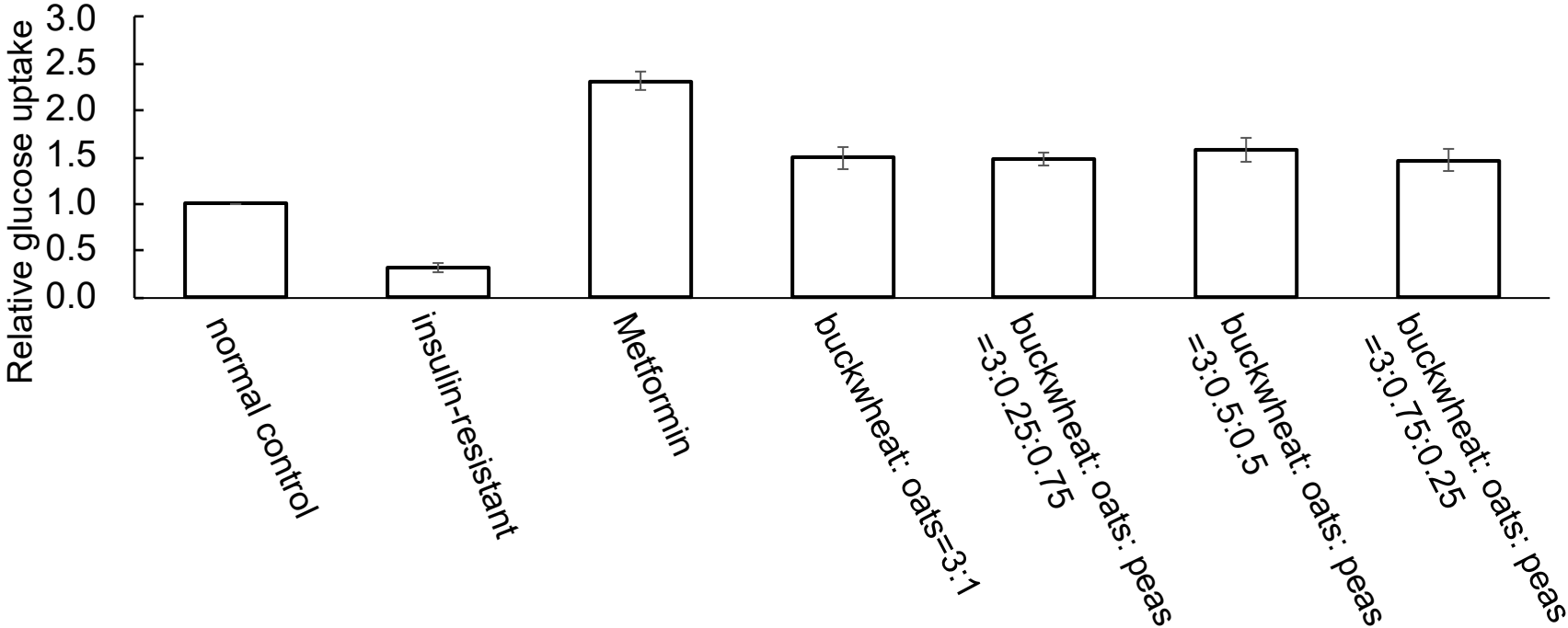

Supplement: Supplementary file 1 [file foods-11-03938-s001.zip › foods-2047910-supplementary.pdf]
